# Natural Antimicrobials Suitable for Combating Desiccation-Resistant *Salmonella enterica* in Milk Powder

**DOI:** 10.3390/microorganisms9020421

**Published:** 2021-02-18

**Authors:** Ahmed G. Abdelhamid, Ahmed E. Yousef

**Affiliations:** 1 Department of Food Science and Technology, The Ohio State University, 2015 Fyffe Court, Columbus, OH 43210, USA; abdelhamid.9@osu.edu; 2 Botany and Microbiology Department, Faculty of Science, Benha University, Benha 13518, Egypt; 3 Department of Microbiology, The Ohio State University, 105 Biological Sciences Building, 484 West 12th Avenue, Columbus, OH 43210, USA

**Keywords:** natural antimicrobials, food additives, *Salmonella enterica*, desiccation resistance, low-a_w_ foods

## Abstract

Some *Salmonella enterica* strains survive well in low-water activity (low-a_w_) foods and cause frequent salmonellosis outbreaks in these products. Methods are needed to overcome such desiccation-resistant *Salmonella* and to improve the safety of low-a_w_ foods. Building on a recent finding, we hypothesized that natural antimicrobial food additives, which are active against cytoplasmic membrane, could overcome this desiccation resistance phenomenon, and thus, sensitize the pathogen to drying and mild processing. Food additives were screened for the ability to cause leakage of intracellular potassium ions; retention of these ions is vital for protecting *Salmonella* against desiccation. Two antimicrobial food additives, carvacrol and thymol, caused considerable potassium leakage from the desiccation-resistant *S. enterica* serovars, Tennessee and Livingstone. Thus, carvacrol and thymol were investigated for their ability to sensitize the desiccation-adapted *S. enterica* to heat treatment. The combined use of food additives, at their minimum inhibitory concentrations, with heat treatment at 55 °C for 15 min caused 3.1 ± 0.21 to more than 5.5 log colony forming unit (CFU)/mL reduction in desiccation-adapted *S. enterica*, compared to 2.4 ± 0.53–3.2 ± 0.11 log CFU/mL reduction by sole heat treatment. Carvacrol was the additive that caused the greatest potassium leakage and sensitization of *Salmonella* to heat; hence, the application of this compound was investigated in a food model against *Salmonella* Typhimurium ASD200. Addition of carvacrol at 200 or 500 ppm into liquid milk followed by spray-drying reduced the strain’s population by 0.9 ± 0.02 and 1.3 ± 0.1 log CFU/g, respectively, compared to 0.6 ± 0.02 log CFU/g reduction for non-treated spray-dried milk. Additionally, freeze-drying of milk treated with high levels of carvacrol (5000 ppm) reduced the population of *Salmonella* Typhimurium ASD200 by more than 4.5 log CFU/g, compared to 1.1 ± 0.4 log CFU/g reduction for the freeze-dried untreated milk. These findings suggest that carvacrol can combat desiccation-resistant *S. enterica*, and thus, potentially improve the safety of low-a_w_ foods.

## 1. Introduction

Low-water activity (low-a_w_) foods are shelf-stable products with an a_w_ of less than 0.85; these include nuts and nut products, honey, spices, dried fruits, and others [1]. In the past two decades, there has been a noticeable increase in the incidence of disease outbreaks linked to low-a_w_ foods such as cereals, nut butter and crackers [2,3,4]. It is estimated that 45% of these outbreaks and the majority of hospitalizations (89%) and deaths (74%), linked to low-a_w_ foods are caused by various *Salmonella enterica* serovars [5]. *S. enterica* may adapt to the dry conditions in these foods and become desiccation-resistant. This adaptation not only allows pathogen cells to survive for long periods in low-a_w_ foods, but also cross-protects these cells against lethal steps in product processing [6].

Despite the progress in assessing the risk of salmonellosis associated with low-a_w_ foods, limited literature addressed strategies to disrupt the desiccation resistance once it is acquired by *S. enterica*. Researchers have proposed increasing process lethality (e.g., elevating heat-treatments) to overcome *Salmonella* in low-a_w_ foods [7]. However, the severity of these treatments is likely to damage the quality of the final product. Hence, our goal was to explore methods to overcome desiccation-resistant *Salmonella* without excessive processing. In a previous study, we found that a newly discovered microbial lipopeptide, paenibacterin, disrupted the desiccation resistance in *S. enterica* and rendered the pathogen sensitive to desiccation [8]. Paenibacterin caused intracellular potassium leakage, increased the cytoplasmic membrane permeability, decreased the biosynthesis of the osmoprotectant trehalose, and downregulated the expression of desiccation-related genes in desiccation-adapted *S. enterica*. Building on these findings, we hypothesized that food additives that are active against bacterial cytoplasmic membranes are likely to compromise the desiccation resistance in *S. enterica*. Such additives would revert the bacterium to its desiccation-sensitive status, and thus, enhance lethality by mild processing technologies. To test this hypothesis, the current study was initiated to (a) screen selected food additives for their ability to compromise the desiccation-resistance in *S. enterica* and (b) assess the ability of a membrane-active additive to inactivate *S. enterica* during milk spray-drying and freeze-drying. Desiccation-resistant *Salmonella* can survive in milk powder and pose food safety risk [9,10,11,12,13]. Therefore, milk powder is an appropriate model to evaluate methods to combat desiccation-resistant *Salmonella*.

## 2. Material and Methods

### 2.1. Preparation of the Test Food Additives

Ten food additives were screened for ability to cause potassium leakage from desiccation-adapted *S. enterica*. Carvacrol and thymol (Sigma Aldrich, St. Louis, MO, USA) were dissolved in 5% dimethyl sulfoxide (DMSO; Sigma Aldrich). Eugenol, trans-cinnamaldehyde, vanillin, diacetyl, and catechin hydrate (Fisher Scientific, Fair Lawn, NJ, USA) were dissolved in 85% ethanol (Fisher Scientific). Benzoic, lactic, and citric acids (Fisher Scientific) were dissolved in sterile water. Five percent DMSO in saline (NaCl; 0.85% *wt*/*vol*), ethanol (85% *vol*/*vol*), or deionized water was used as the additive-free control, depending on the solvent used in a given treatment.

### 2.2. Bacterial Strains

The *S. enterica* serovars used in this study are: (a) *S. enterica* serovar Tennessee E2007000304, a known desiccation-resistant serovar that was isolated originally from peanut butter [14], (b) *S. enterica* serovar Livingstone 1236H (formerly known as Eimsbuettel 1236H [8,15,16]), a peanut butter isolate [17], and (c) *S. enterica* serovar Typhimurium ASD200. The latter is a genetically engineered mutant that was modified (Δ *Salmonella* pathogenicity island [SPI] I and Δ SP2) to an avirulent strain [18]. The strain ASD200 was used only for the spray-drying and freeze-drying experiments. Each serovar was grown in tryptic soy broth (TSB; BD, Sparks, MD, USA) at 37 °C for 18 h before being subjected to desiccation adaptation.

### 2.3. Preparation of Desiccation-Adapted S. enterica Serovars

Desiccation-adapted *Salmonella* serovars were prepared as described in previous studies [8,19] with modifications. Briefly, *S. enterica* serovars were grown overnight in TSB (BD) at 37 °C to a cell density of 10^9^ colony forming unit (CFU)/mL as determined by plating on Tryptic Soy agar (TSA; BD). Cells of the obtained cultures were harvested by centrifugation at 4 °C and 10^4^ × *g* for 5 min and resuspended in fresh TSB. Aliquots (1 mL) of the cell suspension were dispensed in plastic Petri dishes (90-mm diameter; VWR International, Chicago, IL, USA), with a final population of ca. 10^9^ CFU per plate, and air-dried in a biosafety cabinet for 24 h at 22–25 °C under circa 40% relative humidity to prepare desiccation-adapted dry cells. The dried cells were collected and resuspended in saline (0.85% NaCl) to obtain desiccation-adapted cell suspension (DACS).

### 2.4. Potassium Ion Release Assay

**Figure 1:**
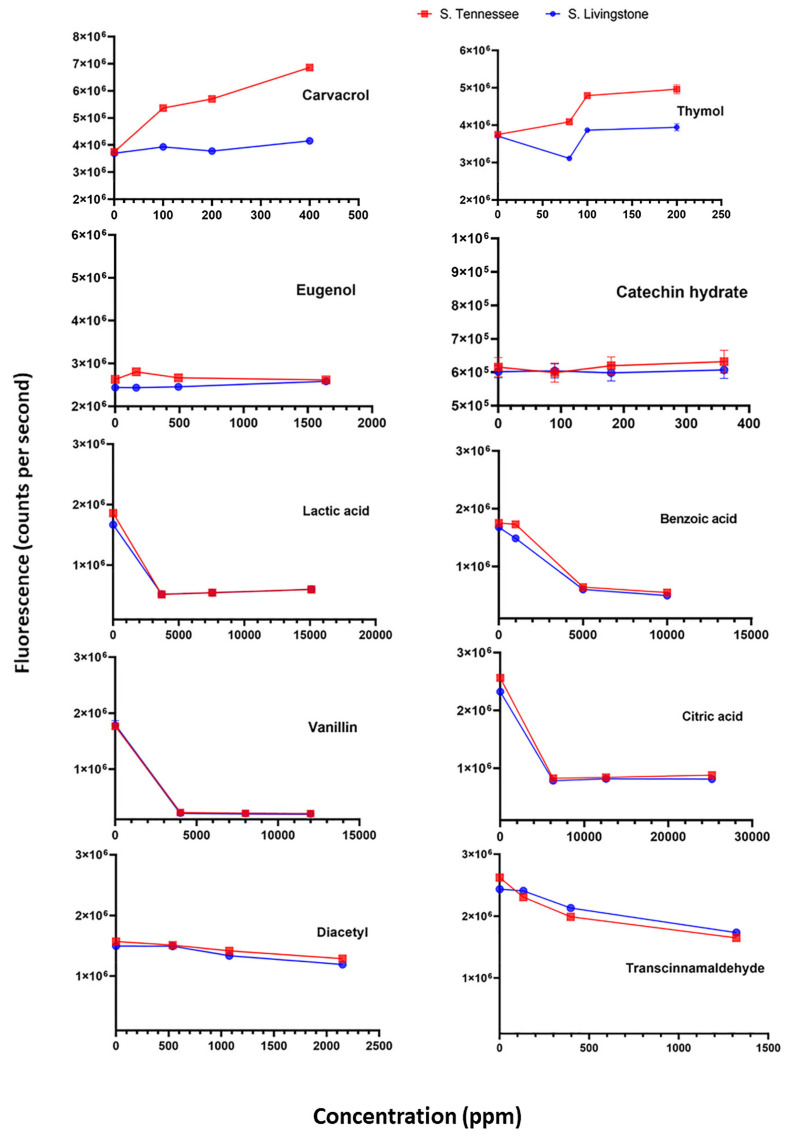
Changes in intracellular potassium ions from desiccation-adapted *Salmonella enterica* serovars in the presence of antimicrobial food additives. Values are the averages of three replicates.

Release of potassium ions was determined using a potassium-binding benzofuran isophthalate probe (PBFI; Invitrogen, Carlsbad, CA, USA) as described previously [8]. Briefly, DACS of *Salmonella* Tennessee and Livingstone were centrifuged and the cell pellets were resuspended in 5 mM HEPES buffer (Sigma Aldrich) supplemented with 5 mM glucose (Fisher Scientific). Aliquots (90 µL) of the resuspended cells were added to wells of a black, nonbinding-surface, 96-well microplate (Corning, Tewksbury, MA, USA). The potassium probe was dispensed to each well at a final concentration of 2 µM before the addition of 10 µL of the food additives, which were prepared as described in a previous section. The food additives were applied at different concentrations (Figure 1) that ranged from sublethal to lethal levels that were reported in previous studies [20,21,22,23,24,25,26] except carvacrol and thymol, which were applied based on their MIC levels determined in the current study. Polymyxin at 10 ppm was used as a positive control. Fluorescence, corresponding to potassium ions concentration, was measured using a microplate reader (Perkin-Elmer, Wellesley, MA, USA) at excitation and emission wavelength of 346 or 505 nm, respectively. Fluorescence measurements were normalized by removing the background fluorescence noise.

### 2.5. Determination of the Minimum Inhibitory Concentration (MIC)

Two food additives, namely carvacrol and thymol, showed significant potassium leakage from *Salmonella* cells. Before these compounds were used for the heat treatment and food application studies, their MICs against *S. enterica* serovars were determined using the broth microdilution method [27]. Briefly, two-fold serial dilutions of carvacrol and thymol, at final concentrations of 800, 400, 200, 100, and 50 ppm, were prepared before 50-µL aliquots of these preparations were dispensed into the 96-well plates (Corning); this was followed by adding equal volume of *Salmonella* Tennessee or Livingstone culture suspension, which was adjusted to OD_600_ of 0.01 in TSB (BD Diagnostic). The MIC of each compound was determined as the lowest concentration, which completely inhibited the visible growth of *S. enterica* after 24 h of incubation at 37 °C.

### 2.6. Treatment of Salmonella Serovars with Additive-Heat Combination

Carvacrol and thymol were assessed for their capability to sensitize *Salmonella* serovars to mild heat treatment at 55 °C for 15 min. Briefly, 5 mL *Salmonella* Tennessee or Livingstone DACSs were treated with carvacrol, or thymol at their MIC (200 and 100 ppm, respectively), while untreated *S. enterica* DACS served as a heated control. Treated and untreated *S. enterica* cells were incubated in a pre-heated water bath at 55 °C for 15 min, plus circa 2.5 min as the sample temperature come-up time. After the thermal treatment, all tubes were removed and held on ice before counts of *Salmonella* survivors were determined using standard plating on TSA (BD).

### 2.7. Inactivation of S. enterica in Milk during Spray-Drying

Carvacrol was selected for applications in milk, as a model food, because of the superior ability of the additive to induce potassium leakage and its ability to sensitize *S. enterica* cells to heat. DACS of *Salmonella* Typhimurium ASD200 was added into 100 mL of liquid milk (Horizon; Organic low-fat milk, obtained for a local supermarket), and a final population of ~10^6^ CFU/mL milk was achieved. Carvacrol at levels of 200 or 500 ppm was added to the milk samples inoculated with *S. enterica* cells, while carvacrol-free inoculated milk samples served as untreated control. Treated and untreated milk samples were dried using a spray dryer (Yamato Scientific Co., Ltd., Tokyo, Japan) at 180 °C and 50 °C for inlet and outlet temperatures, respectively, and a feed flow rate of 10 mL of liquid milk per min. Viable counts of *Salmonella* Typhimurium was determined by plating on the selective agar medium, xylose lysine tergitol–4 (XLT4; BD), before and immediately after spray-drying, and after 1-day storage of the spray-dried milk at 22–25 °C. *S. enterica* populations were determined as CFU/g milk solids. Total milk solids were determined by drying portions of the liquid or spray-dried milk samples on glass fiber pads for 3 min, in a microwave oven, as described previously [28] and the percentage of total solids were calculated from the formula:

(1) $$Total\;solids\;\left(\%\right)\;=\;\left[\frac{\mathrm{weight}\;\mathrm{of}\;\mathrm{dry}\;\mathrm{milk}\;\mathrm{sample}}{\mathrm{weight}\;\mathrm{of}\;\mathrm{wet}\;\mathrm{milk}\;\mathrm{sample}}\right]\;\times \;100$$

### 2.8. Inactivation of S. enterica in Milk during Freeze-Drying

Liquid milk samples (Horizon) were inoculated with *Salmonella* Typhimurium ASD200 to achieve final cell density of 10^7^ CFU/mL before adding carvacrol at 200, 500, or 5000 ppm. The highest concentration tested (5000 ppm) was applied to enable significant reduction of *Salmonella* during freeze-drying. Untreated milk (0 ppm carvacrol) served as a control. All milk samples were frozen at −80 °C for 16–18 h before being dried for 48 h using a freeze dryer (Labconco, Kansas, MO, USA). Freeze-dried milk samples were stored for 2 days at 22–25 °C to measure *S. enterica* survivors during storage. *Salmonella* viable counts were determined as CFU/g milk solids before, and immediately following freeze-drying, and on a daily basis during storage of the freeze-dried milk.

### 2.9. Statistical Analysis

All experiments were performed in triplicate and independently repeated two times, unless indicated otherwise. Bacterial populations were analyzed by analysis of variance (ANOVA) to determine significant differences between treatment groups or Student’s t-test to compare pairs of treatment. The statistical analysis was completed using a statistical software (SPSS; IBM, New York, NY, USA) and a *p* value of <0.05 was considered statistically significant.

## 3. Results and Discussion

### 3.1. Screening Food Additives for the Ability to Disrupt Desiccation Resistance in Salmonella

In a previous study, the membrane-active antimicrobial peptide, paenibacterin, disrupted the desiccation resistance mechanisms and sensitized *S. enterica* serovars to desiccation [8]. Therefore, we explored a similar effect in 10 food additives recognized to exert antimicrobial activity by acting on the bacterial cytoplasmic membrane [29]; these additive are also known to possess the “Generally Regarded As Safe” (GRAS) status. The concentration of additives used encompassed their MIC as reported previously or determined in the current study (Table 1).

The selected food additives were screened for ability to release K^+^ ions from desiccation-adapted *S. enterica* cells. Out of 10 antimicrobial food additives tested, carvacrol and thymol were the most capable of inducing potassium leakage in desiccation-adapted *Salmonella* Tennessee and Livingstone, as shown in Figure 1. Leakage of potassium ions, caused by carvacrol or thymol treatment, may indicate that *Salmonella* has lost one of its important protective factors required for adapting to desiccation [8,19]. If this is the case, treated *Salmonella* becomes sensitive to heat processing and drying stress. Moreover, leakage of intracellular potassium could make the bacterium unable to survive the dry environment during storage of contaminated low-a_w_ food products. These hypotheses were tested in the following experiments.

### 3.2. Sensitizing Desiccation-Resistant Salmonella to Heat Treatment Using Selected Food Additives

**Figure 2:**
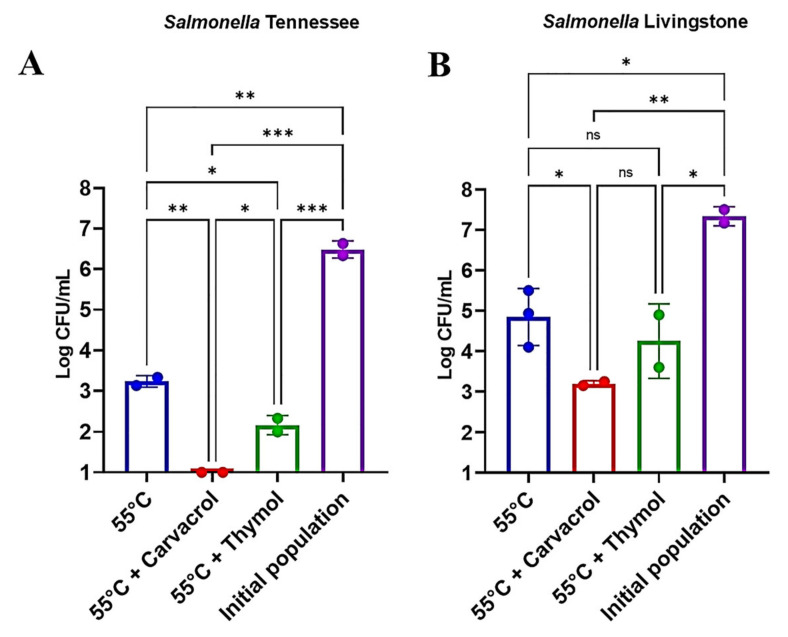
Changes in populations of desiccation-adapted *Salmonella* Tennessee (**A**) and Livingstone (**B**) in response to heat treatment and pretreatment with carvacrol and thymol. Values are averages of three replicates ± standard deviation. Asterisks denote significant differences between averages (* *p* < 0.05, ** *p* < 0.01, *** *p* < 0.001), “ns” = not significantly different.

Carvacrol and thymol were investigated at their MIC (200 and 100 ppm, respectively) in combination with heat treatment at 55 °C for 15 min against desiccation-adapted *Salmonella* Tennessee and Livingstone. Carvacrol, combined with heat treatment, caused reduction of the population of desiccation-adapted *Salmonella* Tennessee by more than 5.5 log CFU/mL, whereas the heat treatment alone caused 3.2 ± 0.11 log CFU/mL reduction (Figure 2A). For *Salmonella* Livingstone, carvacrol combined with heat reduced the pathogen by 4.1 ± 0.21 log CFU/mL compared to the heat treatment alone that caused 2.4 ± 0.53 log CFU/mL reduction (Figure 2B). On the other hand, thymol-heat treatment reduced *Salmonella* Tennessee and Livingstone populations by 4.3 ± 0.22 and 3.1 ± 0.9 log CFU/mL, respectively (Figure 2A,B), compared to the heat treatment alone (3.2 ± 0.11 and 2.4 ± 0.53 log CFU/mL reduction for Tennessee and Livingstone, respectively). These findings indicate that combining food additives with heat is more lethal (*p* < 0.05) than applying heat individually and that serovar Tennessee is more sensitive than Livingstone to this treatment combination. The synergistic effect of carvacrol or thymol with heat could be attributed to the ability of the antimicrobial compounds to damage the cytoplasmic membrane of *Salmonella* cells and further affect the equilibrium of the inorganic ions and induce cell injury [30]. The injury of *Salmonella* by these additives made the bacterium sensitive to heat stress which can damage critical cellular macromolecules, particularly proteins [31]. It is likely that leakage of K^+^ and other osmoprotectans increased intracellular water availability, which, in turn, increases the sensitivity of cellular proteins to heat.

### 3.3. Inactivation of S. enterica in Milk during Spray-Drying

Carvacrol was investigated for the ability to sensitize *Salmonella* Typhimurium ASD200 to spray-drying. The strain ASD200 has been genetically modified through deletion of SPI–1 and SPI–2, and thus, it is suitable for safe use in drying experiments. Carvacrol at 200 or 500 ppm significantly (*p* < 0.05) decreased the viability of the desiccation-adapted strain ASD200 in response to spray-drying, compared to the carvacrol-free control. This effect was observed after 1-day storage of the spray-dried milk at room temperature (Table 2). In contrast, when *Salmonella* populations were determined in the milk powder immediately following spray-drying, no significant difference (*p* > 0.05) was detected between carvacrol-treated or untreated milk. Overall, the results suggest that carvacrol-treated *Salmonella* cells did not implement desiccation resistance in the spray-dried milk during storage, and thus, became dehydration-sensitive. This explanation is supported by a previous finding that pretreatment of desiccation-adapted *Salmonella* with the membrane-active paenibacterin sensitized the cells to subsequent 24 h of desiccation at room temperature [8].

### 3.4. Inactivation of S. enterica in Milk during Freeze-Drying

**Figure 3:**
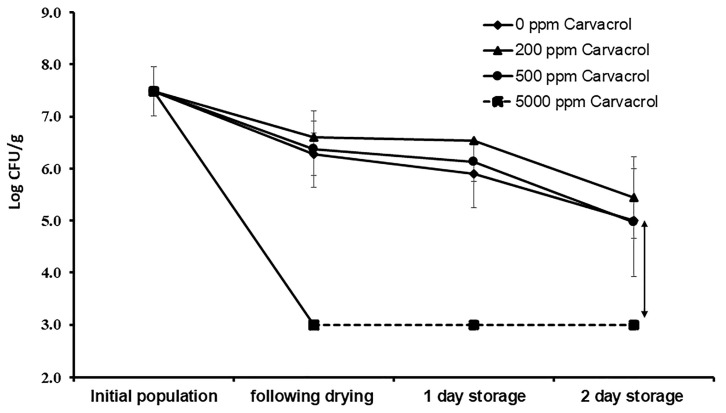
Changes in *Salmonella* Typhimurium populations (log CFU/g milk solids) in the freeze-dried milk processed without or with carvacrol when applied at different concentrations. Values are the averages of three replicates ± standard deviation. The dashed line indicates that *Salmonella* populations were below the enumeration method’s detection limit (i.e., 3 log CFU/g).

Freeze-drying is a dehydration process that is used for preservation of many foods such as fruits, seafood, coffee, and others [32]. In the current study, carvacrol at different concentrations (200, 500, and 5000 ppm) was added into liquid milk to sensitize *Salmonella* Typhimurium ASD200 to the dehydration stress mediated by freeze-drying. Compared to the carvacrol-free freeze-dried milk, samples with carvacrol at 5000 ppm decreased *Salmonella* Typhimurium population below the detection limit of the enumeration method, which was 3 log CFU/g milk (Figure 3). This population reduction was observed immediately following freeze-drying and during the 2-day storage of the dried milk at 22–25 °C. In contrast, treatment with carvacrol at 200 or 500 ppm did not significantly (*p* > 0.05) reduce *Salmonella* levels in the freeze-dried product, compared to the carvacrol-free control (Figure 3).

To determine whether the anti-*Salmonella* activity observed by the combination of carvacrol (at 5000 ppm) and freeze-drying was a synergistic effect between the two lethal factors, a treatment using 5000 ppm carvacrol without freeze-drying was conducted. As shown in Table 3, it was obvious that *Salmonella* Typhimurium survivors were lower following treatment with carvacrol and freeze-drying combination than they were when carvacrol alone was applied. Results from the drying experiments imply that higher concentration of carvacrol (≥10-fold) was required to decrease *Salmonella* populations during freeze-drying, compared to that used in the spray-drying. These findings are plausible considering that spray-drying includes heat; therefore, the bacterium suffered thermal and dehydration stresses, whereas freeze-drying caused dehydration stress only.

Based on these findings, the inclusion of the membrane-active food additive, carvacrol, during drying is advantageous since the compound manipulates *Salmonella*’s physiology in a manner that disrupts the desiccation resistance in pathogen cells. The additive pre-treatment made it easy to inactivate *Salmonella* cells by mild processing such as drying, a process which was also recognized to cause cell death of *S. enterica* by increasing cytoplasmic membrane permeabilization [33]. An additional advantage is that the application of the antimicrobial food additive carvacrol at 5000 ppm with freeze-drying caused more than 4.5 log CFU/g reduction in *S. enterica*. Comparably, a similar reduction in *Salmonella* populations was achieved in non-fat dry milk by applying high temperature (115 °C for 1 h), but this heat treatment impaired the color and gave the powder a burned appearance [34]. Therefore, targeting the cytoplasmic membrane by the membrane-active carvacrol prior to drying is a valuable approach considering it helps overcoming *Salmonella* desiccation resistance and sensitizes the pathogen to the drying processing. The process is also likely to have minimal impact on the milk sensory characteristics, albeit this effect was not tested in the current study.

## 4. Conclusions

The natural antimicrobials, carvacrol and thymol, decreased desiccation resistance in *S. enterica* via induction of intracellular potassium leakage, and this sensitized the pathogen to heat stress. For translating these findings in a food model, the presence of carvacrol during dehydrating liquid milk using freeze- or spray-drying increased the process lethality. Carvacrol is a commercially available GRAS additive, and its use can protect against *S. enterica* contamination and improve the safety of low-a_w_ foods. Future research is needed to determine the effectiveness of carvacrol against desiccation-resistant *S. enterica* in various foods having limited water availability. These are not limited to dehydrated products; it also included frozen foods. Additionally, usage of carvacrol should be optimized to minimize any undesirable sensory changes in treated products.

## Figures and Tables

**Table 1:** The minimum inhibitory concentration (MIC) of the antimicrobial food additives against *S. enterica.*

Category	Food Additive	MIC (ppm)	Source
Plant-derived Biomolecules	Carvacrol	200	Current study
	Thymol	100	Current study
	Trans-cinnamaldehyde	3000	[20]
	Eugenol	512	[21]
	Vanillin	8000	[24]
Weak organic acids	Citric acid	12,624	[23]
	Lactic acid	7552	[23]
	Benzoic acid	>6700 ^a^	[26]
Microbially-derived	Diacetyl	1076	[22]
	Catechin hydrate	181	[25]

^a^ Benzoic acid at more than 6700 ppm was required to cause 50% decrease in *S. enterica* population after exposure for 60 min [26]. Thus, concentrations below and above this level were tested for potassium leakage in the current study.

**Table 2:** Inactivation of *Salmonella* Typhimurium ASD200 during spray-drying of milk with or without carvacrol.

Treatment Time	Moisture Content (%)	*Salmonella* Population (Log CFU/g Milk Solids)	Log CFU/g Reduction *
**Control (0 ppm carvacrol)**			
Time 0 **	85.86	5.6 ± 0.0.05	0.0 ± 0.00
After drying	4.67	5.3 ± 0.21	0.3 ± 0.26
1-day storage	0.25	5.0 ± 0.07	**0.6 ± 0.02a ^#^**
**Carvacrol (200 ppm)**			
Time 0	85.86	6.1 ± 0.51	0.0 ± 0.00
After drying	4.67	5.9 ± 0.17	0.2 ± 0.34
1-day storage	0.25	5.2 ± 0.53	**0.9 ± 0.02b**
**Carvacrol (500 ppm)**			
Time 0	85.86	6.6 ± 0.02	0.0 ± 0.00
After drying	4.67	6.1 ± 0.02	0.5 ± 0.00
1-day storage	0.25	5.3 ± 0.13	**1.3 ± 0.10c**

* The reduction in *Salmonella* ASD200 populations was calculated in reference to the initial population before spray-drying. ** Refers to the initial population of *Salmonella* ASD200 before starting the spray-drying process. *Salmonella* ASD200 population reductions, highlighted in bold, indicate the highest lethal effects of carvacrol treatments ^#^ Data followed by different letters indicate significant difference (*p* < 0.05).

**Table 3:** Changes of *Salmonella* Typhimurium populations in carvacrol-treated milk followed with or without freeze-drying.

Treatment	Log CFU/g Total Solids of Milk			
	Initial Population	Day–0	1-Day Storage	2-Day Storage
Carvacrol (5000 ppm) + Freeze-drying	7.5 ± 0.5	<3 ^a^	<3 ^a^	<3 ^a^
Carvacrol (5000 ppm)	7.5 ± 0.3	5.4 ± 0.02	3.9 ± 0.6	4.9 ± 0.4

^a^ Below the enumeration method’s detection limit (i.e., 3 log CFU/g).

## Data Availability

Not applicable.
